# Approach and Fork Insertion to Target Pallet Based on Image Measurement Method

**DOI:** 10.3390/s26010154

**Published:** 2025-12-25

**Authors:** Nobuyuki Kita, Takuro Kato

**Affiliations:** TICO-AIST Cooperative Research Laboratory for Advanced Logistics, National Institute of Advanced Industrial Science and Technology (AIST), Tsukuba 305-8560, Japan; takuro.katou@aist.go.jp

**Keywords:** logistics, pallet, automated forklift operation, fork insertion, wide field-of-view image, calibration

## Abstract

Previously, we proposed a navigation method based on image processing to move to the front of a pallet that can be detected in the field of view. We also proposed an image-processing method to estimate the inclination angle when the pallet is inclined at a pitch with respect to the running surface, such as a pallet loaded on a truck. In this study, we improved the robustness of the existing method so that the series of operations from approach to fork insertion can be realized stably without being affected by the environment. Furthermore, a series of operations from approach to fork insertion was realized by an automatic forklift (Autonomous Guided Forklift, AGF) in an indoor laboratory space simulating a warehouse.

## 1. Introduction

With the increase in the volume of distribution, aging, and a decrease in the working population, the automation of work at distribution sites is an urgent issue. This paper introduces a final report on the realization of automated insertion of a fork into a pallet, which is indispensable for unloading a load from a truck in the work of a forklift and essential for distribution.

Automated forklift navigation can be classified into two types. One is navigation for pallet transportation between two spatially separated points, not only indoors but also outdoors, such as in warehouses. This has not been specialized for pallet transportation, and many studies have been conducted on general autonomous mobile vehicle (AGV) navigation [1,2]. Regarding in-warehouse movement at logistics sites, a method of embedding an index in the environment, such as a guide rail [3,4], was proposed in the past; however, more recently, methods exclusively based on the SLAM method [5,6,7] have been proposed.

However, when the forklift and pallet to be transported are spatially close to each other, a method has been proposed in which the position and orientation of the pallet are measured by a sensor mounted on the forklift, and navigation is performed while the relative position and orientation relationship between the forklift and pallet is measured.

Since our main objective is to automate the task of unloading pallets mounted on trucks that arrive by means of forklifts waiting outdoors, we take this approach.

The following operations are required to insert the fork into the pallet on the truck:

1. Movement of the forklift from the standby position to the front of the pallet loaded on the truck that has arrived.
2. Adjustment of the fork position and posture, and forward movement of the forklift.

To automate these operations, it is necessary to measure the positional relationship between the forklift and pallet, and the position and posture between the fork and pallet. Several measurement methods that use various sensors have been proposed to realize these operations. We aimed to realize these operations using a wide field-of-view image measurement, and the reason for selecting a wide field-of-view image measurement is elaborated upon in Section 2.

**Figure 1:**
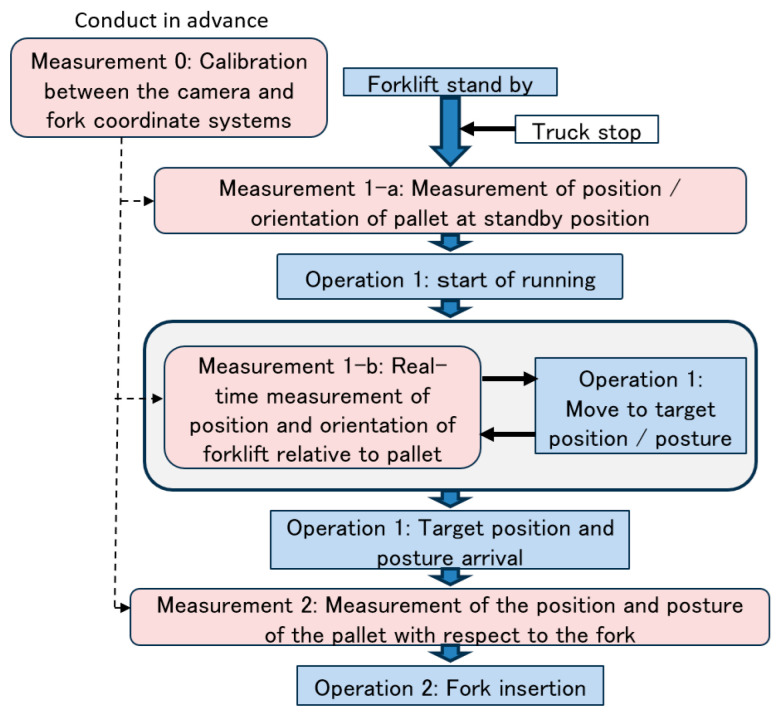
Relationship between forklift operation and image measurement.

The relationship between the forklift operation and image measurement is shown in Figure 1. Operation 1 required the following two image measurements:

Measurement 1-a. Measurement of the position and orientation of the pallet in the standby position.

Measurement 1-b. Real-time measurement of the position and orientation of the traveling forklift relative to the pallet.

Operation 2 required the following:

Measurement 2. Measurement of pallet position relative to the fork. In addition, because the loading surface of a truck may be inclined at a pitch with respect to the running surface, it is necessary to measure the inclination angle.

In addition, because the image measurement data needs to be converted from the camera coordinate system to the forklift coordinate system for Operation 1, and from the camera coordinate system to the fork coordinate system for Operation 2, Measurement 0, the calibration between these measurements, is essential.

We developed a method for performing these measurements using only wide-field images obtained using a single fisheye camera [8,9,10,11,12,13]. Refs. [8,9,10] proposed methods for measuring the position and orientation of a pallet using a camera coordinate system. Refs. [12,13] proposed a method for measuring the position and orientation of a forklift truck in real time and a navigation method for moving the forklift truck to the front of the pallet based on this measurement. Ref. [11] proposed an image-processing method for estimating the inclination angle of a pallet loaded onto a truck when the pallet is inclined in pitch with respect to the running surface. An outline of each method is presented in Section 3.

This paper describes the robustness of an existing method, which was developed to realize a series of operations from the approach to fork insertion in a stable manner, regardless of environmental conditions. In addition, a series of operations from the approach to fork insertion were realized by AGF in an indoor experimental space simulating an actual place.

## 2. Related Works

Several studies have been conducted to realize automated pallet transfers. There have been several proposals to detect pallets from the background in sensor data [14,15,16,17,18], and to measure the position and orientation of the detected pallets [19,20,21,22,23,24]. However, most of these target pallets are placed on a flat surface parallel to the floor surface, such as a floor or shelves, and only target the orientation around the yaw axis perpendicular to the floor surface. In recent years, many studies have used deep learning to improve not only the detection performance but also the accuracy of measuring the position and orientation (6 DOF) of pallets in the sensor coordinate system [25,26,27,28,29,30]. Ref. [19] has measured pallets using two types of distance sensors, as well as the bed and empty space of trucks loading pallets.

For fork insertion, research has been conducted to navigate the forklift truck to the front of the pallet based on the results of position and orientation measurements [31,32,33,34,35]. Refs. [31,33,35] have proposed a position and orientation measurement method and a path generation method for moving toward the front of a pallet. Refs. [32,34] have discussed position and orientation measurements, path generation, and travel control; however, the experimental results were limited to measurement evaluation.

As in this study, some studies have targeted the operation up to the insertion of the fork and conducted operation tests using a forklift [36,37,38,39]. In [36], the position and orientation of the pallet were measured by detecting a marker attached to the pallet or an edge in contact with the floor in front of the pallet from the images of two cameras fixed at separate positions, and a trajectory was generated by a 5-dimensional polynomial. While updating the traveling position and orientation by odometry, if the error becomes large, navigation is performed to the fork insertion preparation position by performing image measurement and trajectory regeneration. Finally, the fork is inserted into the pallet by moving straight forward by a specified distance. Ref. [37] realized the position and orientation measurement of a pallet of unknown size based on straight-line detection from scan data by 2D-LRF, and realized fork insertion by odometry-based movement. In [38], the detection of the pallet group, measurement of the position and orientation, navigation to the fork insertion position, and fork insertion were mainly realized using RGB-D camera images after reaching the pallet storage position. Pallet detection uses a deep learning method for RGB images. Based on the distance information equivalent to the detected image region, the rectangular surface of the foot of the pallet is detected, and the position and orientation of the pallet are measured under the constraints of its geometric shape and arrangement, which are generated by the A* algorithm for the pallet located closest to the position where the fork can be inserted. Navigation is then performed, and the fork is inserted under the pallet by visual feedback. The operation is similar to that in this study, but the difference is that the pallet placed on the floor is the target, and a distance image is used. In [39], the pallet position and attitude are measured by the distance data obtained by 2D-LiDAR; after the route by a straight line and circular arc is generated, the travel is controlled along the route by the LiDAR-Servo, and the fork insertion is realized.

Some studies include remote transport [40,41]. Approximately 30 years ago, before the advent of distance sensors, Ref. [40] performed remote movement by visual recognition of a marker placed in the environment. After approaching the pallet, the position and orientation were measured by detecting a hole as a dark area from the image of a camera placed at the fork root. It was possible to insert the fork within a distance of 2–4 m and an orientation of plus or minus 10°, although the details of the method and experiment are unknown. Ref. [41] realized a more general operation. Specifically, two pallet storage locations were set at known positions without visibility from the outdoor forklift standby area, and the task of moving a group of pallets placed appropriately on one side to the other side was automated. Navigation between the storage locations was adopted using a variant of the SLAM method. The detection of pallets and measurement of their position and orientation were performed using a DL-based method [28], in which the forklift was moved to the fork insertion position by visual servoing, and the insertion was controlled by an internal sensor. It is of interest to evaluate performance by comparing the total work execution time with the work time of experienced operators. However, because there was no description of the experiments and success rates for each operation, a comparison with this study was not possible. In addition, there was no mention of fork insertion into an inclined pallet.

The only studies and experiments on fork insertion into an inclined pallet were by [42,43]. In this method, the point group in front of the load mounted on the pallet was acquired using a distance sensor, the inclination of the load was measured by applying a plane surface, and the inclination of the pallet was obtained from the relationship between the front surface of the load and the pallet. This method is different from the one used in this study, in that a distance sensor was used, and it could not be applied when no load was mounted on the pallet.

Among the studies mentioned above, refs. [20,21] explicitly described the coordinate transformation from the camera coordinate system to the fork coordinate system, etc. Ref. [20] performed the calibration between the camera coordinate system and the fork coordinate system using Zhang’s method [44], and Ref. [21] performed the independent calibration only for 1 DOF out of six DOF between RGB-D and the fork. In addition to our work, Ref. [36] proposed his own calibration method, which was based on the mobile camera-space manipulation (MCSM) method, an extension of the camera-space manipulation (CSM) method [45].

Table 1 summarizes the approaches to the image measurement methods from the literature for [Measurement 0], [Measurement 1-a], [Measurement 1-b], and [Measurement 2] (as described in this paper), which discuss travel control for fork insertion. For [Measurement 0], those who proposed an original method were marked with a circle, and those who did not were marked with a cross. For [Measurement 1-a], those who proposed an original method were marked with a circle. For [Measurement 1-b], those that proposed an original method are marked with a circle, those that were not described in the paper are marked with a cross, and those that used the same method as Measurement 1-a were marked with a triangle. For [Measurement 2], those that considered pallet inclination were marked with a circle, and the others were marked with a cross.

The literature referenced in this section uses cameras, range sensors, or both; however, none of the studies used wide-field images as inputs, as we did. We chose wide-field images as the inputs for the following reasons. As the front of the pallet must always be within the measurement range from the start to the end of the movement, the wider the measurable range of the sensor, the wider the pallet position through which the cargo can be handled. There is a method to widen the measurable range using multiple sensors or by controlling the position and orientation of the sensors. However, there are problems with the arrangement of the sensors and control accuracy. Therefore, we adopted a fisheye camera with a field of view of more than one hemisphere and fixed it to the backrest of the AGF facing forward. In this work, the processing target was the front-facing camera image of the 360° camera, Ricoh Theta S, which consisted of two cameras, the front and rear cameras; the front-facing camera had a 218° angle of view and 640 × 640 pixel resolution.

## 3. Overview of Wide-Field Image-Processing Methods

The method outlined in [8,9,10] was used to estimate the position and orientation of the pallet in the camera coordinate system for [Measurement 1-a]. According to [8,9,10], by assuming that the shelf or floor surface on which the pallet is placed is horizontal, the orientation of the pallet is estimated by assuming that the degree of freedom is only the angle around the vertical axis (Yaw angle).

### 3.1. Overview of [Measurement 0]

**Figure 2:**
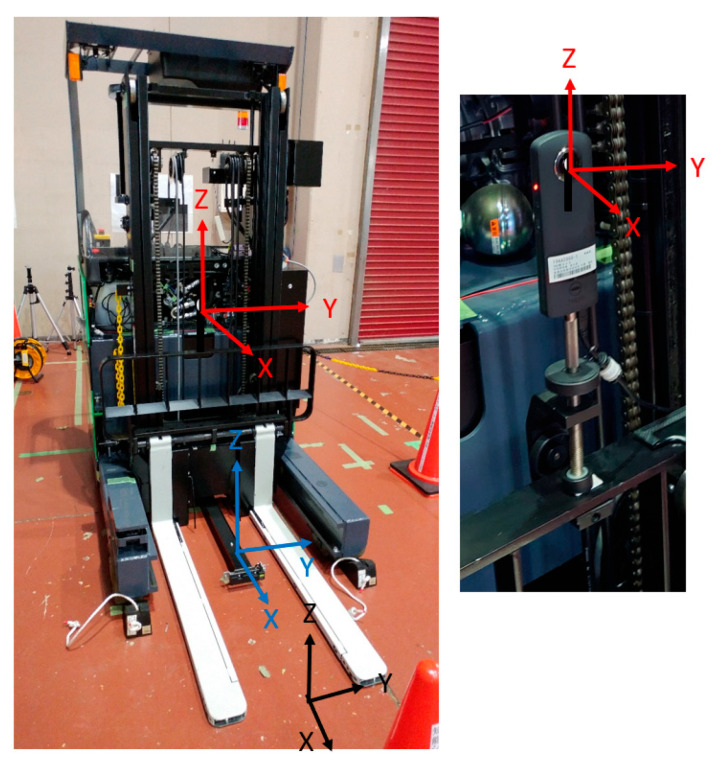
Camera coordinate system (red), forklift coordinate system (blue), and fork coordinate system (black).

Figure 2 shows the camera coordinate system (red), forklift coordinate system (blue), and fork coordinate system (black). The camera coordinate system is a right-hand system with the optical center as the origin, the optical axis direction as the *X*-axis, and the upward direction of the image as the *Z*-axis. The fork coordinate system is a right-hand system with the fork’s upper surface as the reference plane, which is placed on the reference plane corresponding to the midpoint of the line segment connecting the left and right fork tips. The direction of the line segment on the reference plane is the *Y*-axis, and the direction perpendicular to and upward from the reference plane is the *Z*-axis. The camera was fixed to a backrest using a simple jig (Figure 2). The posture was adjusted such that it was as parallel to the fork coordinate system as possible. However, because the camera housing was small, there would inevitably be several differences. Because the backrest and fork were integrated, the camera and fork coordinate systems exhibited a rigid relationship. The calibration error of the posture relationship between the two coordinate systems directly affected the accuracy of the pitch inclination estimation of the pallet, as will be described later on in this paper.

The forklift truck coordinate system is a right-hand system with the origin at the midpoint of the two front wheels, the *X*-axis in the direction of travel on a plane parallel to the floor surface passing through the origin, and the *Z*-axis vertically upward. The backrest and fork move up and down, tilt, and back and forth with respect to the forklift, but are fixed at the basic position and orientation during travel. The basic position/orientation is the position/orientation at which the fork reaches, and the tilt is set to zero (the vertical position is an arbitrary position). The fork coordinate system is parallel to the forklift truck coordinate system. As the two postures coincide, the positional relationship between them can be accurately measured using a tape measure. Therefore, the calibration target is the position/orientation relationship between the camera and fork coordinate systems.

In a previous study, the author proposed a method [46] to obtain the direction vector of a group of three-dimensional parallel straight lines from panoramic images. Because three-dimensional straight lines are projected as curves in panoramic images, the straight lines are detected via local edge-point detection (using the Sobel operator) and the Hough transformation devised for panoramic images. According to this method, the direction vector of a group of parallel straight lines in the camera coordinate system can be estimated if two or more parallel straight lines in three-dimensional space can be detected in a panoramic image (equirectangular image). Hereafter, this method will be referred to as the Orientation Vector (OD) method.

**Figure 3:**
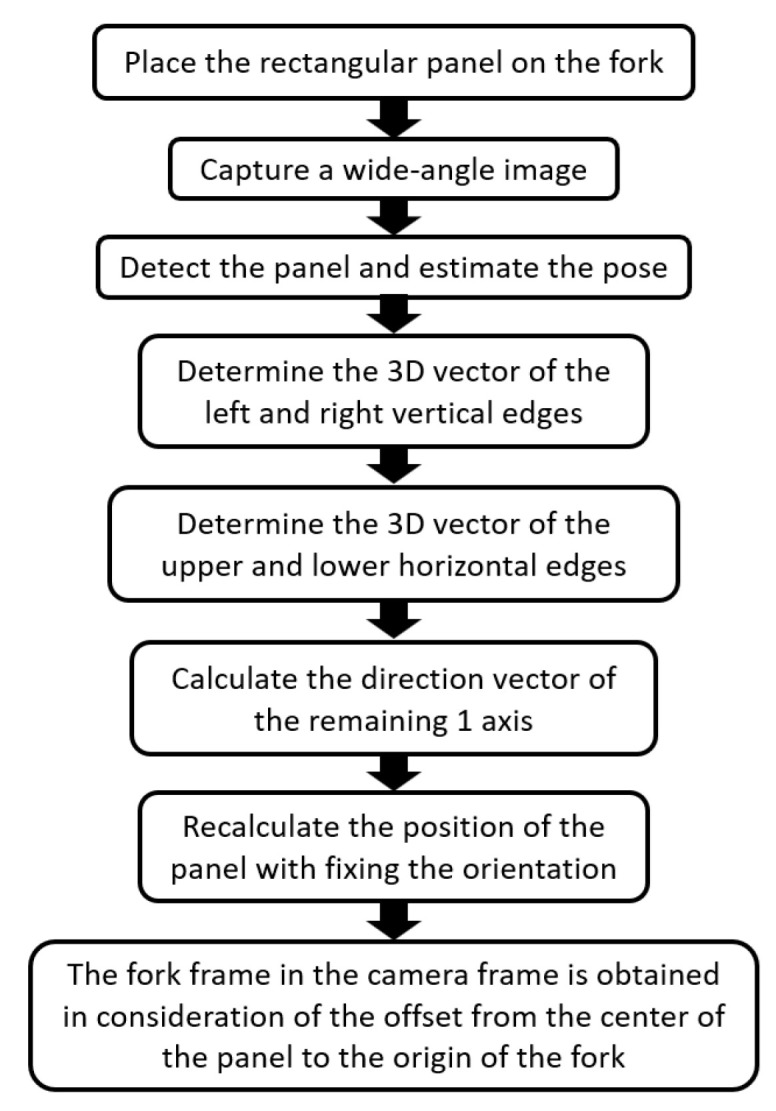
Flow of calibration processing.

**Figure 4:**
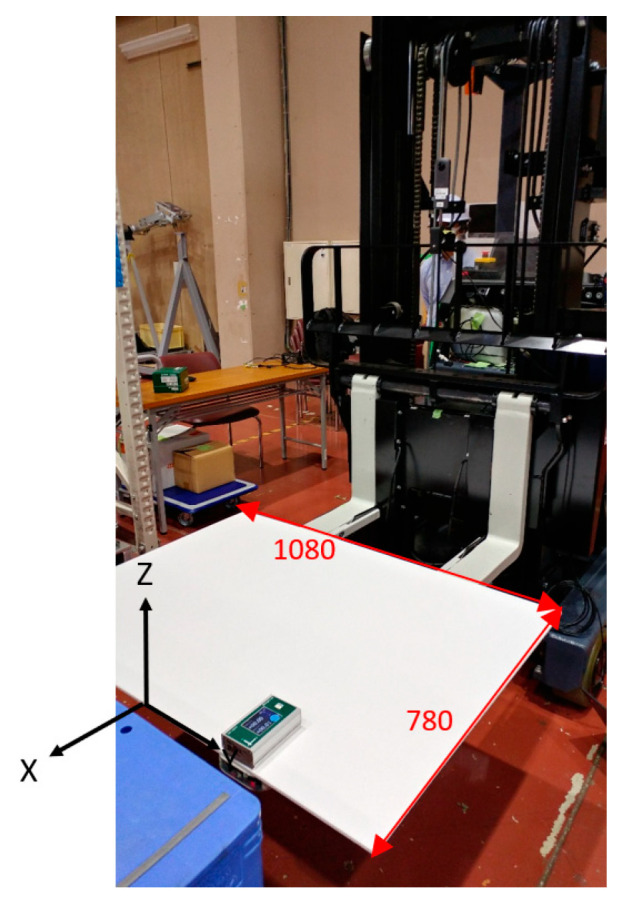
Set up for calibration image.

**Figure 5:**
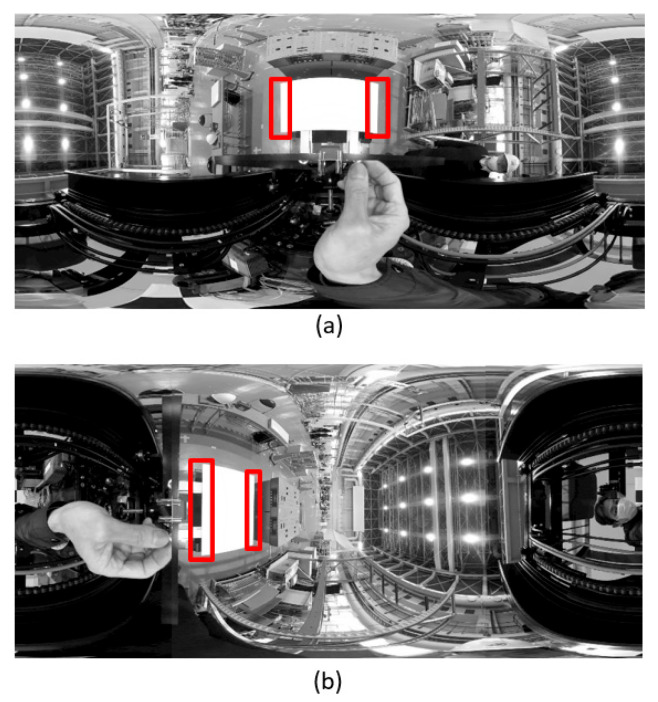
Applying OD method to panoramic images. (**a**) Obtaining the vertical direction vector of the panel. (**b**) Obtaining the horizontal direction vector of the panel. Red frames show the vertical edge detection area.

Figure 3 illustrates the flow of the calibration process. A rectangular panel of known dimensions was placed on the fork such that the center of the front lateral edge coincided with the origin of the fork coordinate system, and the vertical and horizontal edges were parallel to the X- and Y-axes of the fork coordinate system (Figure 4). The panel was detected from the image using the methods outlined in [8,9], and the position and orientation of the panel in the camera coordinate system were estimated. At this point, errors existed in the orientation estimation. Next, the wide-field image was converted into a panoramic image with the *X*-axis of the camera coordinate system as the central axis (Figure 5a), and the vertical direction vector of the panel was obtained by applying the OD method to the region where the left and right vertical edges of the panel existed. Next, the wide-field image was converted into a panoramic image with the *Y*-axis of the camera coordinate system in the vertical direction (Figure 5b), and the horizontal direction vector of the panel was obtained by applying the OD method. Orientation errors were eliminated by obtaining the direction vector of the remaining axis from the vertical and horizontal direction vectors of the panel and updating the orientation of the panel in the camera coordinate system. After fixing the orientation of the panel in the camera coordinate system to the updated orientation, the methods of [8,9] were applied again to increase the accuracy of the position estimation of the panel in the camera coordinate system. Finally, the position and orientation of the fork coordinate system in the camera coordinate system were obtained by considering the offset from the center of the panel to the origin of the fork coordinate system according to the position and orientation of the panel in the camera coordinate system.

### 3.2. Overview of [Measurement 1-b]

**Figure 6:**
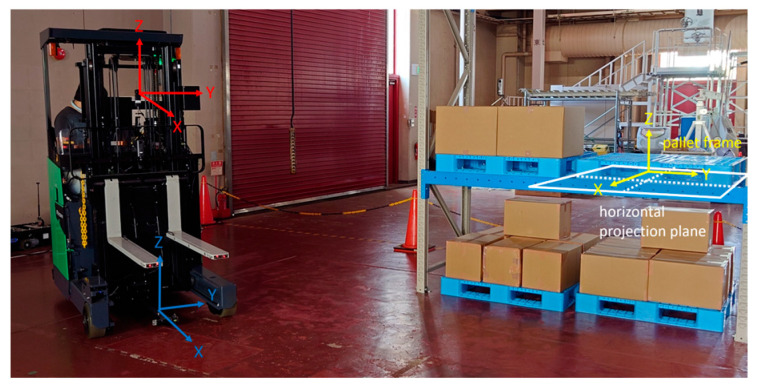
Camera coordinate system (red), forklift coordinate system (blue), and pallet coordinate system (yellow) during running and horizontal projection plane.

It was assumed that the running surface was flat, and the pallet was placed on the running surface or on a parallel plane (the pallet may be inclined by pitch). Figure 6 shows the relationship between the coordinate systems during the operation. The pallet coordinate system was set such that the center of the front surface of the pallet to be handled was the origin, the front direction was the *X*-axis, and the vertical upward direction was the *Z*-axis. That is, the XY planes of the moving camera, forklift, and pallet coordinate systems are regarded as parallel to each other.

To control the forklift, its XY position and yaw angle (posture around the *Z*-axis) in the pallet coordinate system were measured in real time. As the position and orientation of the forklift at the starting point have already been measured, the position and orientation after the movement can be estimated from the amount of movement and deformation of the image of the pallet between the input images before and after traveling. For this purpose, horizontal and vertical projection plane images were used.

**Figure 7:**
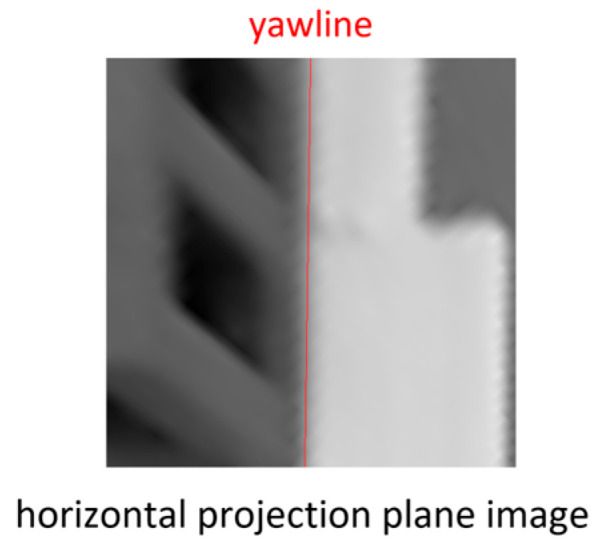
Horizontal projection plane image and the yawline.

**Figure 8:**
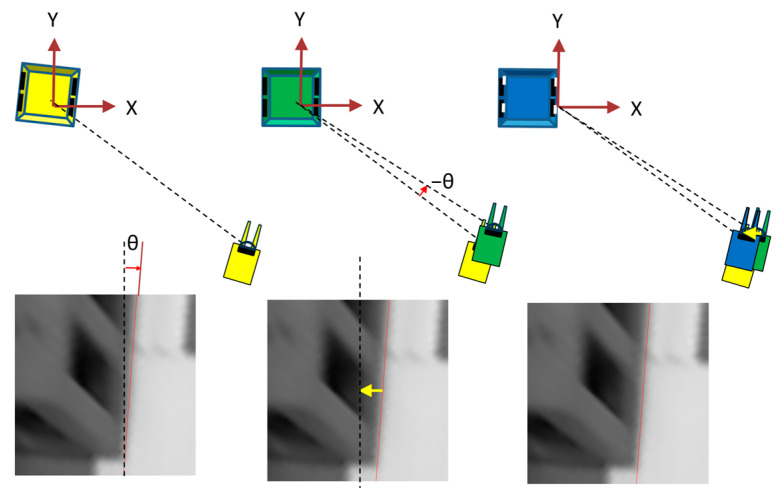
Updating XY position and yaw orientation from the shift amount of the yawline. The lower three images are horizontal projection plane images created on the basis of the estimated position and orientation before moving from the input image after moving, and the yawline deviates from the centerline. The upper diagram shows the update process of the estimated position and orientation. Yellow represents the forklift and palette image in the position and orientation estimation before updating, green represents the forklift and palette image after updating based on the deviation (θ) of the yawline inclination, and blue represents the forklift and palette image after updating based on the horizontal deviation (yellow arrow) of the yawline.

As shown in Figure 6, a horizontal projection plane image was created by back-projection from the input image on a horizontal projection plane obtained by moving the XY plane of the pallet coordinate system parallel to the height of the bottom of the pallet; for example, each pixel has a side of 4 mm and a size of 500 pixels × 500 pixels (Figure 7). Because the image of the lower edge of the front surface of the pallet was a straight line that extended long in the vertical direction, the edge points were extracted, and a Hough transformation was performed to detect it. Hereafter, the detected straight line is referred to as the yawline. If the camera was not moving, the yawline was a vertical line that passed through the center of the horizontal projection plane image in the left–right direction. If the camera moved in the opposite direction, the yawline shifts. Subsequently, the XY position and yaw orientation can be updated based on the amount of shift, as shown in Figure 8.

**Figure 9:**
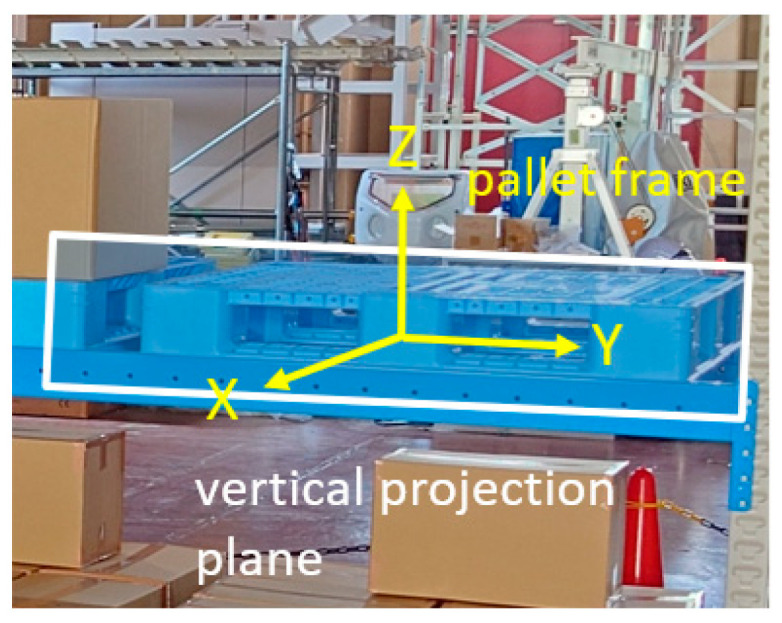
Vertical projection plane.

**Figure 10:**
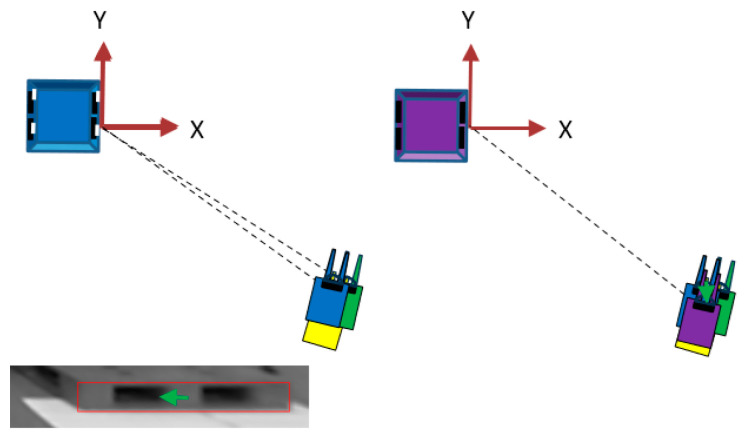
Updating the Y-position by the length corresponding to the amount of lateral shift in the center of the pallet front view. The bottom image is a vertical projection plane image created from the input image after movement on the basis of the blue estimated position and orientation updated in Figure 8. The front view of the palette is horizontally offset from the center (green arrow). The forklift and palette images updated by the distance equivalent to this offset are shown in purple.

The vertical projection plane image shown in Figure 9 was projected onto a vertical projection plane set on the YZ plane of the pallet coordinate system. If the camera was not moving, the front view of the pallet was projected onto the center of the vertical projection plane image, and the shift from the center reflected the amount of movement. Therefore, the center of the front view of the pallet was detected by template matching using the front model of the pallet, and the camera position was updated in the Y-direction by a length corresponding to the amount of the lateral shift (green arrow in Figure 10). This completes the update of the camera position and orientation.

### 3.3. Overview of [Measurement 2]

**Figure 11:**
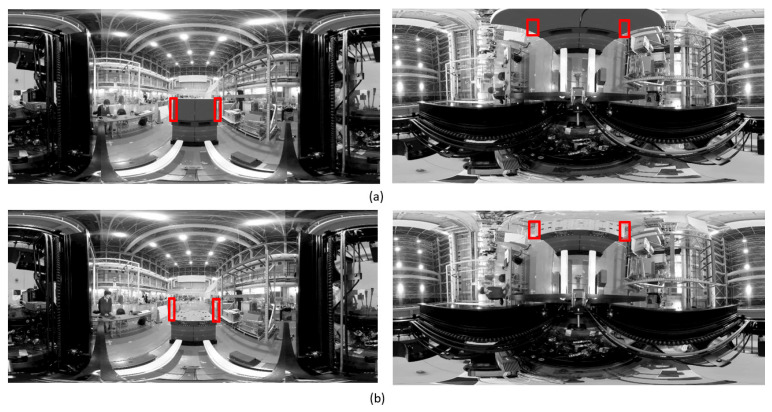
Applying the OD method to panoramic images for the measurement of pitch angle. (**a**) When there is a load, the straight line is hidden and is not detected in the detection area in the **right** figure. (**b**) When there is no load, the straight line due to the load is not detected in the detection area in the **left** figure. Red frames show the vertical edge detection area.

**Figure 12:**
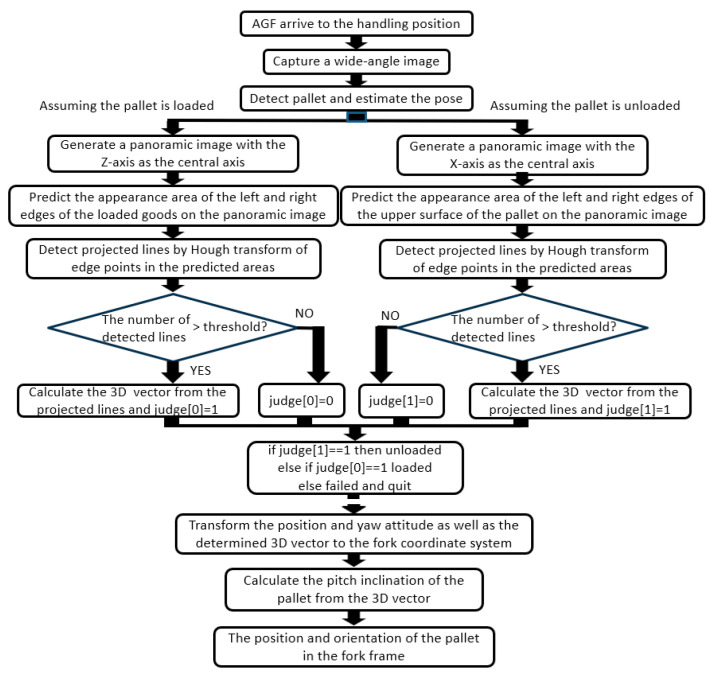
Overall procedure of pitch angle measurement.

As the position and orientation of the forklift in the pallet coordinate system were known when the forklift reached the front of the target pallet via automatic travel, the position and orientation of the pallet in the camera coordinate system could also be derived. However, as the measurement was based on the assumption that the pallet was placed parallel to the traveling surface, the pitch angle with respect to the floor surface was unknown. As shown in Figure 11, the pitch angle was measured using the OD method from the longitudinal edge of the loaded goods when the pallet was loaded, and from the left and right edges of the upper surface of the pallet when the pallet was not loaded. As it was unknown whether the pallet was loaded, a panoramic image centered on the *Z*-axis and a panoramic image centered on the *Y*-axis were created from an input image. An attempt was made to detect the edge of a load by the former and to detect the edge of the upper surface of a pallet by the latter, and the presence or absence of a load was determined depending on whether a straight line could be detected in a detection area, as shown in Figure 12.

## 4. Robustness of Line Detection

The core technology of our original methods includes the following:

[A] Straight-line detection, such as the perimeter of a rectangular panel for [Measure 0]

[B] Yawline detection for [Measure 1-b]

[C] Detection of lines on both sides of the top surface of the pallet or lines extending up and down the load [Measure 2].

**Figure 13:**
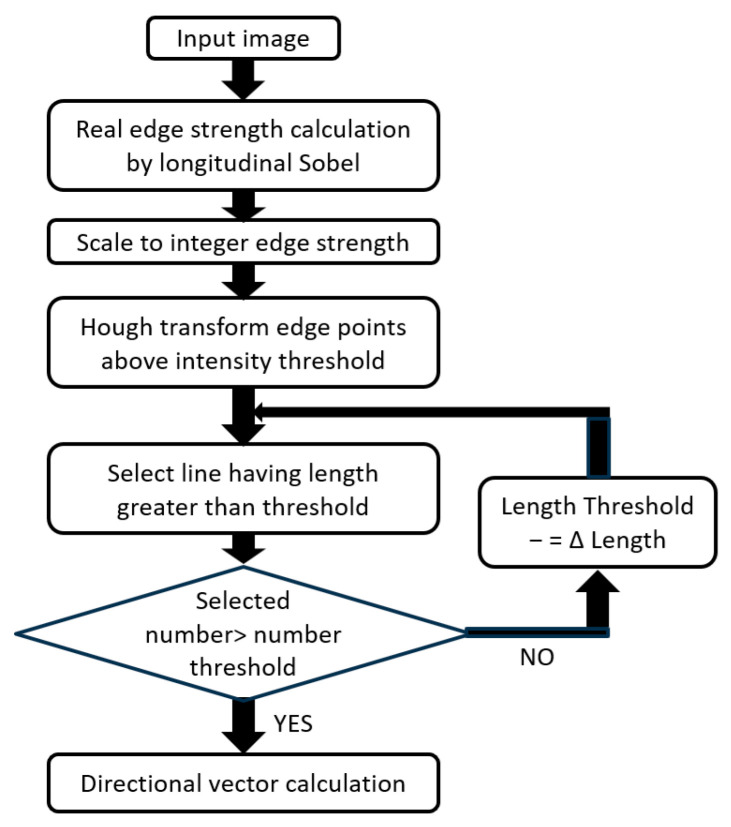
Algorithm based on length of line on image as selection criterion.

All these methods detected straight lines from an image. All these techniques were based on a method for detecting three-dimensional straight lines extending in the vertical direction in a panoramic image [46], which the author developed as a zenith correction method for panoramic images. The algorithm for zenith correction detected artificial vertical lines present in buildings and indoors on a panoramic image and geometrically determined the zenith direction from selected representative straight lines. Longer three-dimensional straight lines projected on the panoramic image contributed to stable and accurate determination; therefore, they were selected based on the length of the image. All the core techniques [A], [B], and [C] of the proposed method were implemented using an algorithm based on the length of a line in the image as a selection criterion, as shown in Figure 13.

**Figure 14:**
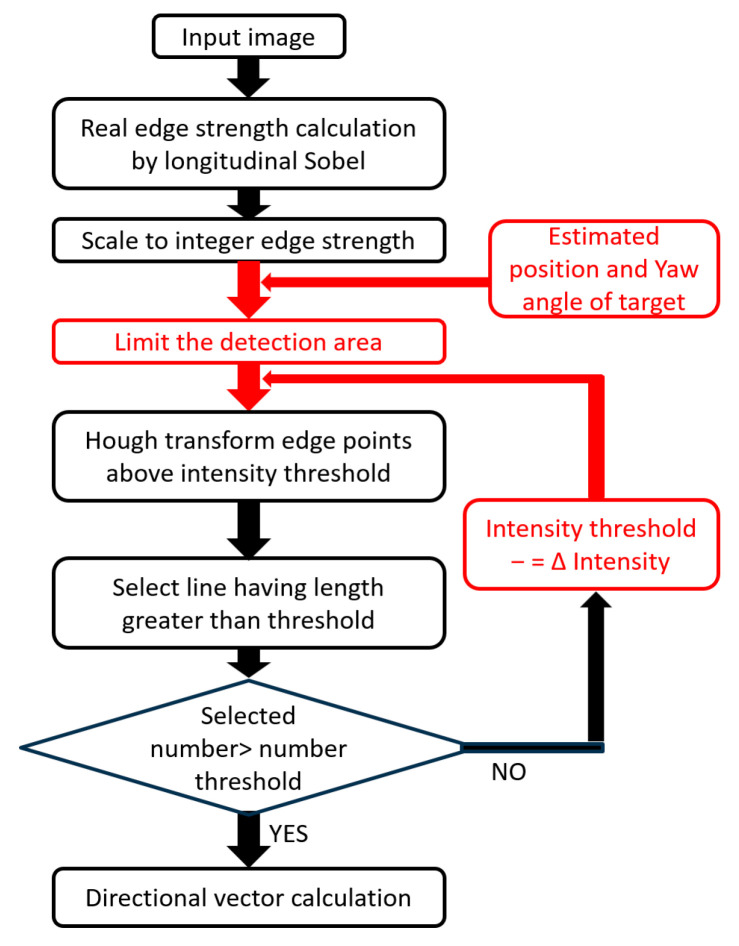
Algorithm based on edge strength on image as selection criterion.

To further strengthen the robustness of [A], [B], and [C], the characteristics of the straight lines detected from the image were arranged as shown in Table 2. Based on this consideration, it was found that the length of the straight line to be detected could be limited for all the core technologies based on the position of the pallet and the yaw pose estimation (Measurement1-a) performed prior to line detection. Therefore, we decided to use edge strength as the adoption standard instead of length as the selection standard based on the detection-candidate straight lines. Therefore, it was revised as shown in Figure 14. That is, the threshold value of the length of the straight line was fixed, for example, 80% of the limited area’s length. Subsequently, the edge-strength threshold value for the Hough transformation was gradually reduced until the desired number of straight lines satisfying the length threshold value was obtained.

**Figure 15:**
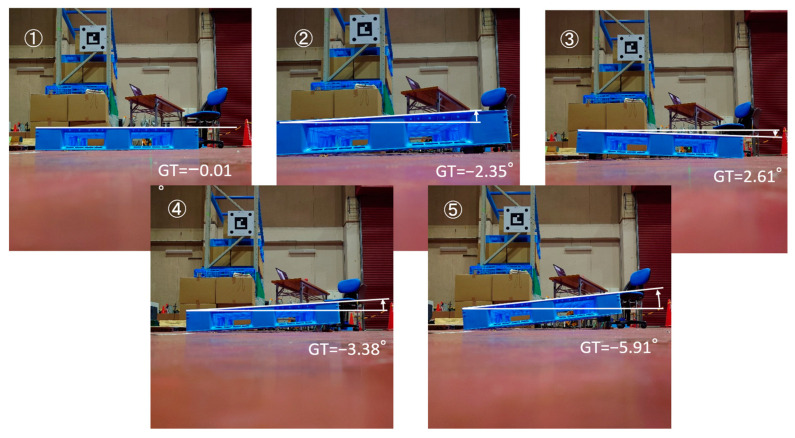
Five types of unloaded pallets with different inclination angles.

To measure the pallet inclination angle [Measurement 2], the measurement results before and after the improvement were compared. Table 3 lists the angle measured by the inclinometer (GT) and the inclination angle obtained by image measurement (Estimated) for the five types of unloaded pallets with different inclination angles (Figure 15). The difference between GT and Estimated included the calibration error between the camera coordinate system and the fork coordinate system. With this calibration error as an offset, it was calculated as the average of the difference between the true value and the estimated value. When the difference between (Estimated–Offset) and GT was compared, the maximum absolute value decreased significantly from 0.56° before the improvement to 0.13° after the improvement.

## 5. Robustness of [Measurement 0]

### 5.1. Issues

**Figure 16:**
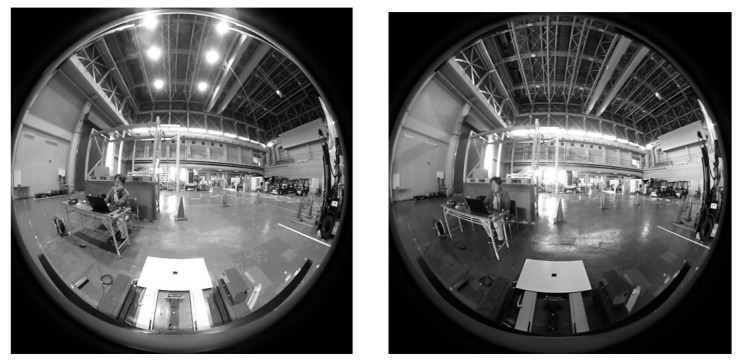
Images for calibration taken by changing only ON/OFF state of ceiling light.

In the conventional method, there was a problem that the calibration result slightly varied depending on the lighting condition and background change. Figure 16 shows an image for the calibration obtained by changing only the ON/OFF state of the ceiling lighting. The calibration results, that is, the position and orientation of the target in the camera coordinate system, were as follows: The unit of the position was meters, and the orientation was expressed in AngleAxis, which began with an angle and is in degrees.

position = 1.14431 0.0151662 −1.08514

orientation = 1.92608 0.572591 −0.764234 −0.296793

when it was ON, but

position = 1.15054 0.0148959 −1.09401

orientation = 2.4994 0.856975 −0.515339 −0.00444753

when it was OFF. The posture changed by approximately 0.6°.

**Figure 17:**
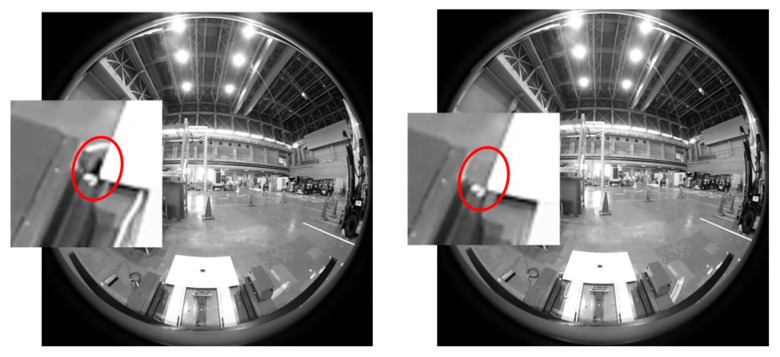
Images for calibration showing only the difference between the presence and absence of a car stop, which appears as a small black piece in the red circle on the left image.

Figure 17 is an image for calibration showing only the difference between the presence and absence of a car stop in the background of the lower left corner of the rectangular panel, and the calibration result is as follows:

Position = 1.14456 0.0149857 −1.08532;

Orientation = 1.8914 0.583252 −0.758233 −0.291375;

With the car stop, but

Position = 1.14381 0.0140624 −1.08768;

Orientation = 2.66621 0.871687 −0.489274 −0.0277815;

Without the car stop. The attitude has changed by nearly 0.8°.

**Figure 18:**
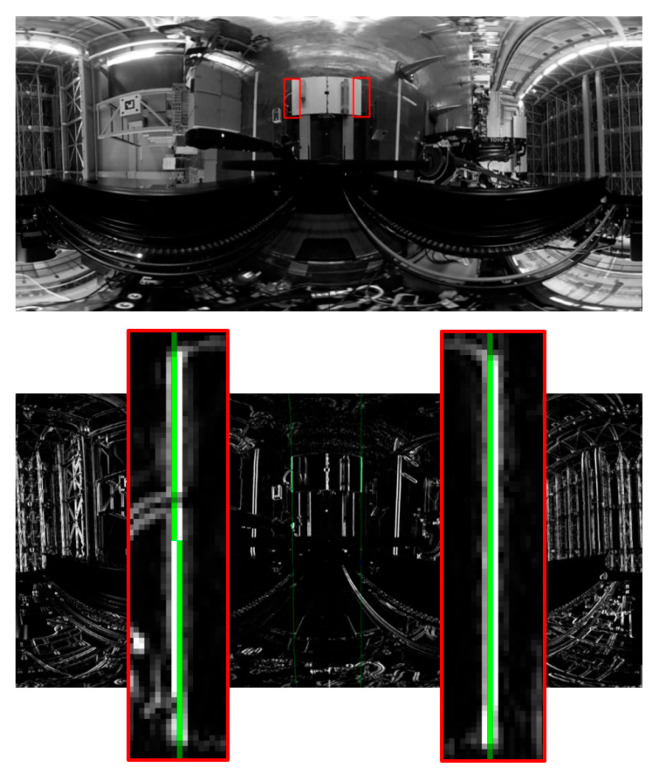
Left and right vertical edges of the panel are close to a straight line on the panoramic image. The **upper** image is a panoramic image created around the *X*-axis, and the **lower** image is the edge image and an enlarged view of the detection area. Red frames show the vertical edge detection area and green lines show the detected straight lines.

Since the left and right vertical edges of the panel at the time of calibration were close to parallel to the *X*-axis of the camera coordinate system, they were close to a straight line on the panoramic image as shown in Figure 18, so there was also a problem that the measurement of the inclination of the straight line became unstable due to the Hough transform from only the edge points in the detection area.

In particular, the pitch inclination of the fork coordinate system with respect to the camera coordinate system directly affected the pitch angle measurement of the pallet and determined the success or failure of fork insertion. Therefore, the following measures were taken to make the system robust.

### 5.2. Improving the Central Axis of Panoramic Images

When a straight line parallel to the central axis of the panoramic image approached a straight line on the panoramic image, the measurement of the inclination became unstable. Therefore, the image was converted into a panoramic image by using an axis with a depression angle to the camera’s optical axis as the central axis, after which the depression angle was subtracted from the measured value.

### 5.3. Use Rectangular Chess-like Panels

**Figure 19:**
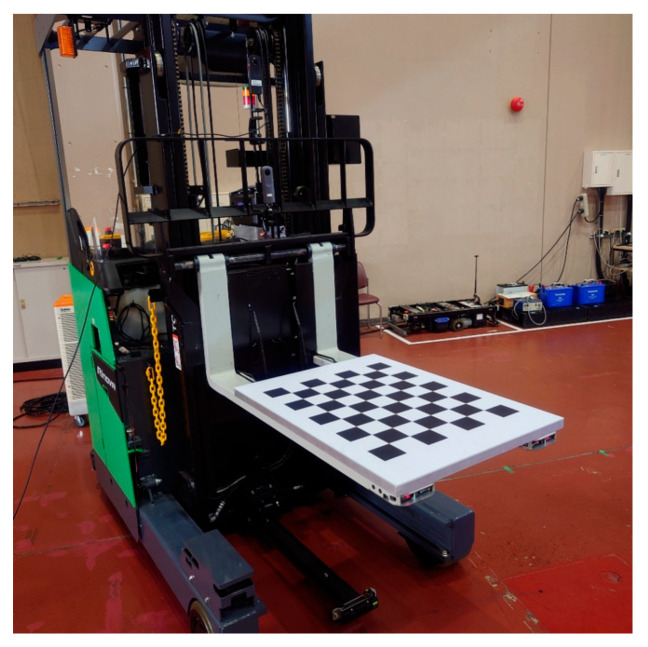
Rectangular panel with chess pattern as calibration target.

Because the method for obtaining the direction vector from each of the two vertical and horizontal straight lines of the plain rectangular panel was limited, a rectangular panel with a chess pattern was selected as the calibration target (Figure 19).

### 5.4. Results

**Figure 20:**
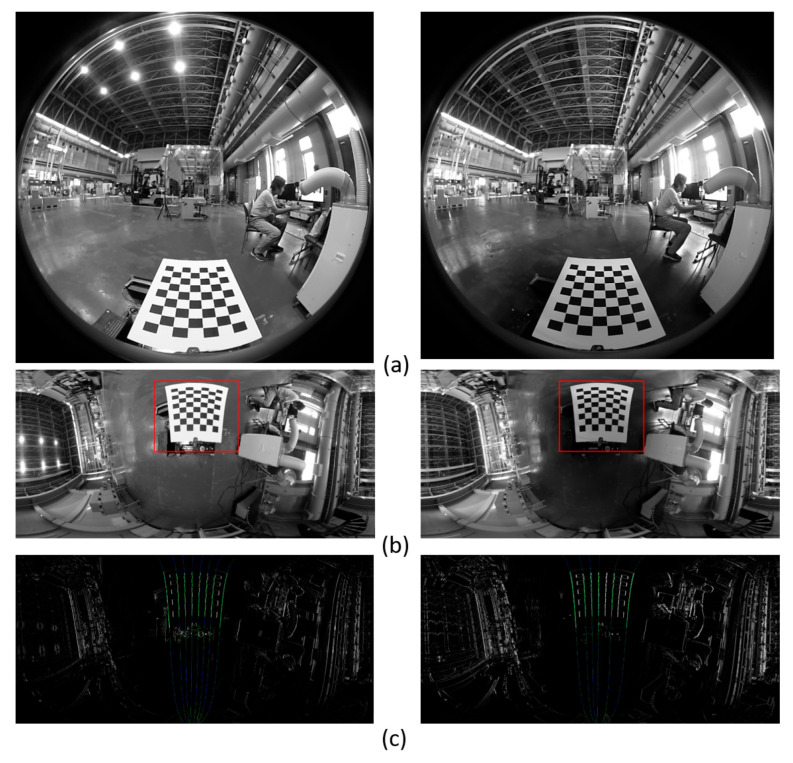

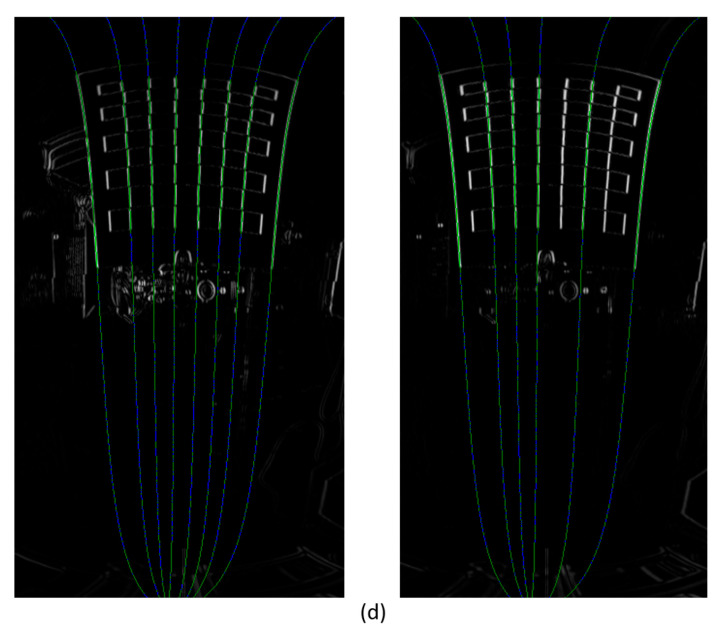
Calibration images and processing results obtained by changing only the ON/OFF state of the ceiling lighting. (**a**) Input image; (**b**) panoramic image created with the axis where the *X*-axis is tilted as the central axis and the vertical edge detection area (red frame); (**c**) edge image and a detected straight line (green and blue line); and (**d**) enlarged view of (**c**), in which a three-dimensional straight line is detected as a curve on a panoramic image, and a plurality of straight lines are detected regardless of the difference in illumination.

Figure 20 shows the calibration images and processing results obtained by changing only the ON/OFF state of the ceiling lighting. Calibration results showed the following:

Position = 0.24573 −0.0135096 −0.644342;

Orientation = 1.66259 0.842323 −0.41232 −0.347109;

With the ON state, and

Position = 0.250193 −0.0139664 −0.651508;

Orientation = 1.74777 0.807334 −0.484901 −0.336278;

With the OFF state. The difference in the measured values of the posture with and without lighting was 0.1° or less, which is negligibly small.

## 6. Robustness of [Measurement 1-b]

### 6.1. Yawline Detection Considering Edge Orientation

**Figure 21:**
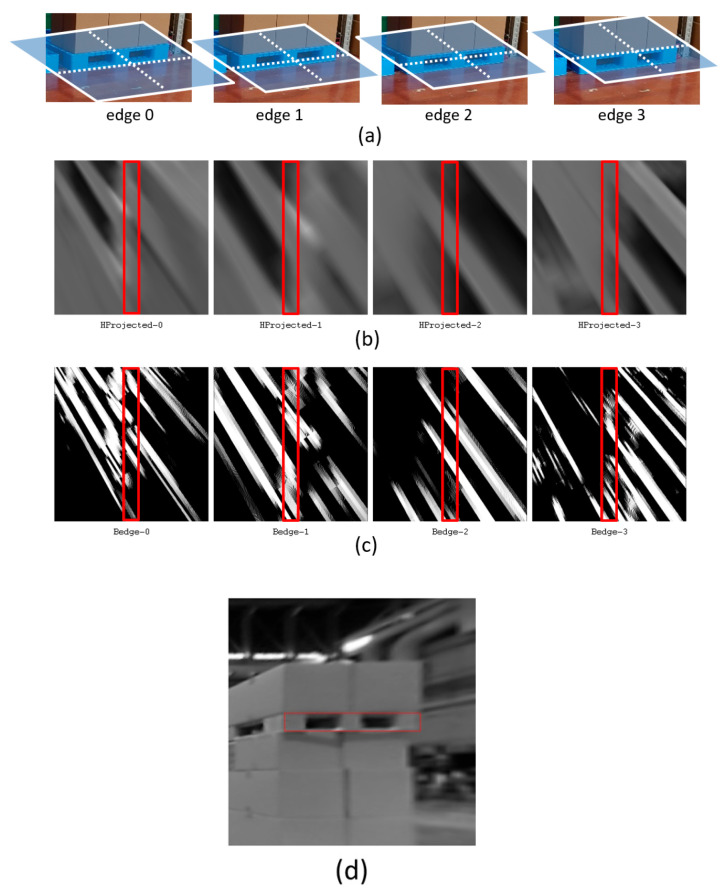
Horizontal line (considered to be optimal) is selected from edge intensity of central region shown by red farames. (**a**) Horizontal projection planes at the position of four yawline candidates, (**b**) horizontal projection plane images, (**c**) edge images and (**d**) vertical projection plane image just for the sake of clarity.

In a previous study [13], with the aim of continuously and stably continuing yawline detection during traveling, a horizontal projection plane image (Figure 21b) was created at the position of four yawline candidates, as shown in Figure 21a, at the start of traveling. A horizontal line considered to be optimal was selected, and at the same time, a scale value for converting the real-number edge intensity into the integer edge intensity was determined from the edge intensity (Figure 21c) of the central region (within the red frame). In the example shown in Figure 21d, edge1—that is, the lower edge of the hole—was determined to be optimal with a scale of 6.36389.

However, for example, edge0 and edge1 were close to each other, so there was a situation in which they were confused while driving. Therefore, the attribute of whether the real edge strength after applying the Sobel was positive or negative was considered. Consequently, although the edge strength changed, the direction of the shade change was not reversed; for example, edge0 and edge1 were not confused.

### 6.2. Compute Scale for Each Input Image

Because the viewpoint position of the input image changed every moment while the vehicle was running, and the edge intensity also changed accordingly, the scale value calculated at the start of driving often did not provide an edge image suitable for detection. Therefore, the scale value was automatically determined for each image frame based on the distribution of the real edge intensities in the detection area.

## 7. Robustness of [Measurement 2]

### 7.1. Compute Scale Adaptively

**Figure 22:**
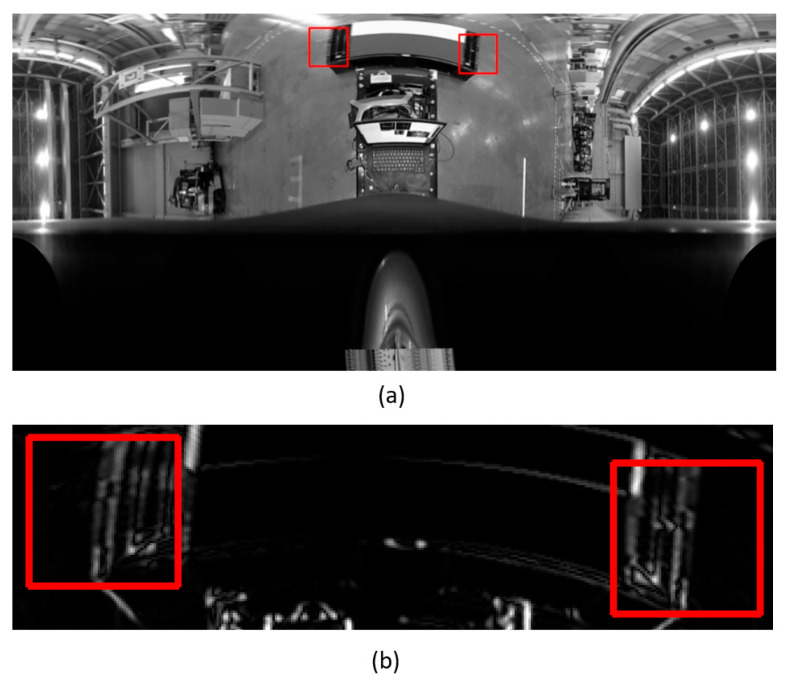
Straight line cannot be detected in the detection area by the conventional method. (**a**) Panoramic image and detection area (red frame). (**b**) Enlarged view of the edge image.

**Figure 23:**
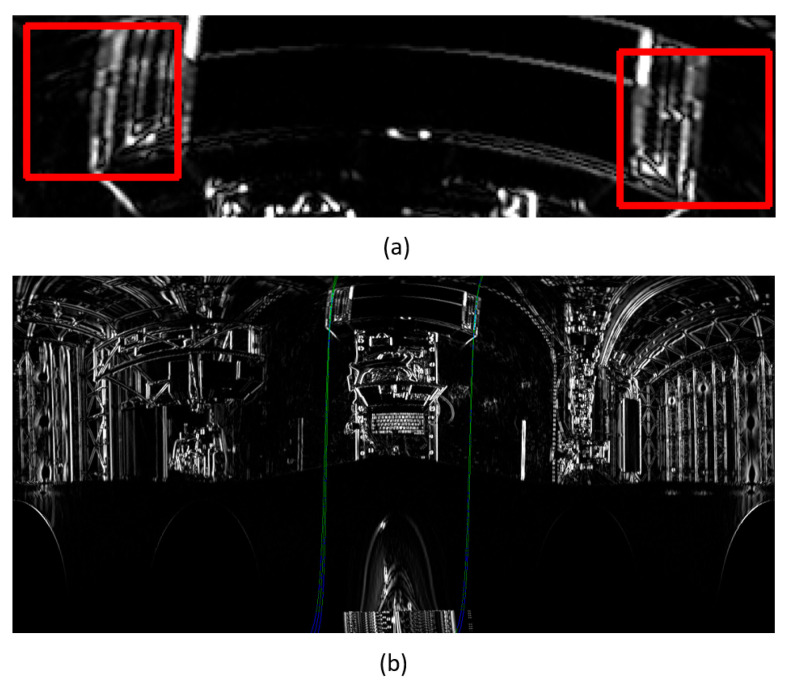
Straight line can be detected, and pitch inclination angle is successfully estimated. (**a**) Enlarged view of an edge image created after improvement and detection area (red frame). (**b**) Detected straight line (green line).

In the conventional method of calculating a scale for converting a real-number edge intensity to an integer, when a pixel with a locally high edge intensity exists in the detection area, as shown in Figure 22, the scale was estimated to be small; consequently, a straight line could not be detected in the detection area. Therefore, the scale was calculated so as not to be affected by pixels with locally high edge intensity. As a result, a straight line could be detected, as shown in Figure 23, and the pitch inclination angle was successfully estimated.

### 7.2. Improving the Central Axis of Panoramic Images

**Figure 24:**
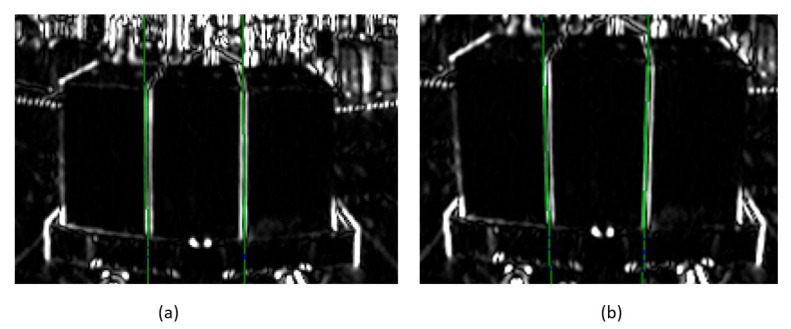
Changing the central axis of panoramic images greatly decreases the error of pitch angle measurement. (**a**) shows detected straight line (green line) before the improvement and (**b**) shows detected straight line (green line) after the improvement.

In the example shown in Figure 24a, where the inclination angle of the pallet was measured by the conventional method, there seemed to be no problem in straight-line detection; however, there was an error of approximately 1.37° from the measurement by the inclinometer. Therefore, in the creation of the panoramic image, assuming that a load was present, the central axis was not set to the *Z*-axis but to an axis obtained by inclining the *Z*-axis forward by 10° (Figure 24b), and the error from the measurement by the inclinometer was greatly improved to approximately 0.08°.

### 7.3. Improvement of Measurement Timing

This was not a problem with the image measurement method, but there was an issue with the measurement timing. Previously, the pitch inclination angle was measured based on the input image obtained immediately after receiving a signal from the forklift that had reached the goal. However, the forklift moved forward by several centimeters to several tens of centimeters because of inertia, even after the driving force was set to 0. Because this distance varies randomly due to its relationship with the running surface, it was unpredictable. Therefore, the time at which the forklift stops was determined by comparing the measurement positions in the preceding and following frames on the image measurement side, and the pitch inclination angle was measured in the image immediately thereafter.

## 8. Position and Attitude Control Experiment of Forklift and Fork Based on Image Measurement

Before implementing the robustness improvement described in this paper, the algorithm was verified in a well-maintained indoor environment as introduced in Section 4 of Kita24, and the vehicle was able to reach the goal position by starting from a position of 2 m in each of the X- and Y-directions in the pallet coordinate system. In Kita25, verification was carried out in an environment close to the field in terms of the influence of lighting and background. It was possible to reach the position where the fork can be inserted by starting travel from the position of 2 m in each of the X- and Y-directions, with the pallet placed on the floor as the target. However, image measurement failed during travel when a pallet placed on a shelf was the target. In addition, when travel started from the required position of 3 m in each of the X- and Y-directions at the pallet site, yawline detection was mistaken at the first turning, and travel could not be continued.

As a result of the efforts made to overcome this situation (Appendix A), it was possible to carry out the experiment described below, that is, the experiment in which a pallet placed on a shelf was handled in a near-field environment, the traveling start position was set at a distance of approximately 3 m in each of the X- and Y-directions of the pallet coordinate system, and the forklift travelled and inserted the fork.

Using a reach-type forklift truck (Toyota AGF Rinova 8AFBR15 manufactured by Toyota Industries Corporation, Kariya, Japan), an automatic pallet-handling control experiment was conducted by applying the improved image measurement method to an actual forklift truck. The forklift truck was equipped with a MicroAutoBox II (an in-vehicle real-time control unit) manufactured by dSPACE, Paderborn, Germany and was modified to obtain vehicle body sensor values, such as vehicle speed, steering angle, and fork height, as well as to output control signals to drive wheels and fork operation actuators.

A laptop PC (Intel Core i7@2.60 GHz, 32 GB RAM) and an omnidirectional camera (Ricoh Theta S manufactured by Ricoh Company, Ltd., Tokyo, Japan) mounted on the backrest of the forklift truck were used for image measurement. The camera images were imported into the PC via USB and processed at 5 Hz. The obtained image measurement values were sent to the MicroAutoBox II via UDP and reflected in the traveling and handling controls of the forklift truck.

### 8.1. Forklift Movement Control Based on Image Measurement

**Figure 25:**
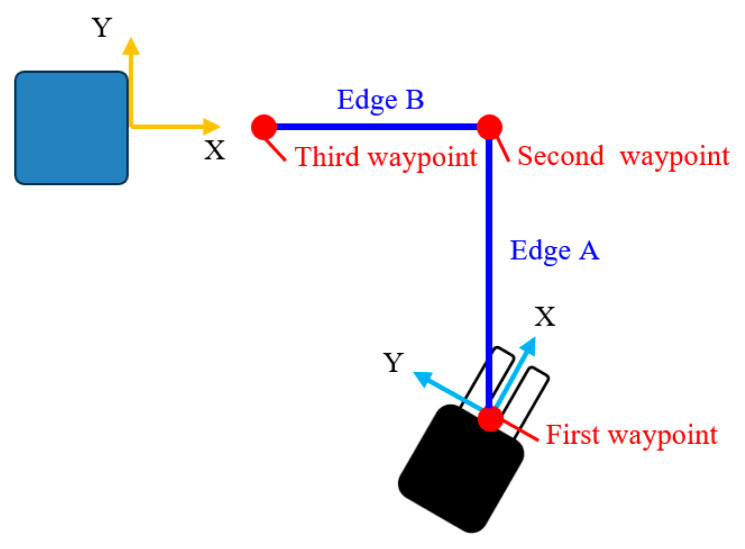
Three waypoints to guide the forklift to the front of the pallet.

The forklift was guided to the front of the pallet based on the XY and yaw positions of the forklift in the pallet coordinate system obtained by the pallet measurement. Three waypoints were set, as shown in the route diagram in Figure 25. First, the vehicle was turned at the first waypoint (initial position) such that its posture was parallel to the *Y*-axis. The vehicle was then moved to the second waypoint along Edge A, parallel to the *Y*-axis. After reaching the second waypoint, the vehicle was turned 90° again and moved to the third waypoint along Edge B.

The turning motion at the first and second waypoints was performed by P control, which input the difference between the target angle and the current angle by rotating the drive wheels forward under the condition that the steering angle was fixed at 90°. To follow Edge A, P control that inputs the lateral (X-direction) position deviation between the vehicle body and the edge was used. The Pure Pursuit method [47] was applied to follow the Edge B, with a look-ahead distance of 0.8 m when the distance between the vehicle body and the route was 0.03 m or more, and 2.0 m when the distance was less than 0.03 m. The image measurement values of the XY position and yaw posture of the forklift in the pallet coordinate system obtained at 5 Hz were interpolated using vehicle wheel odometry to update the self-position at 100 Hz. The forward speed of the forklift was 1 km/h.

### 8.2. Fork Insertion Control

**Figure 26:**
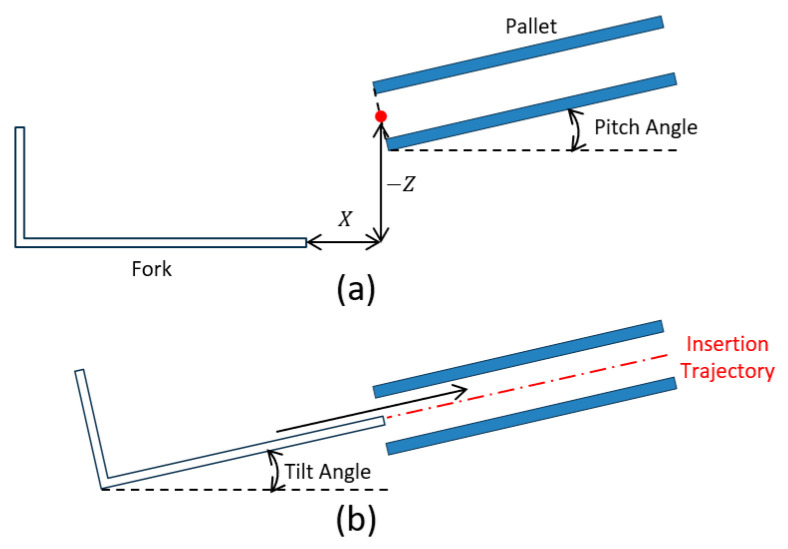
Two step operation of the fork insertion. (**a**) First stage. (**b**) Second stage.

After the forklift stopped at the front of the pallet, the position and angle of the fork were adjusted by two-stage control according to the XZ position of the front center of the pallet in the fork coordinate system and the inclination angle measured from the image, and the fork was inserted into the inclined pallet. In the first stage, the operation started from the state shown in Figure 26a, and the fork was controlled such that the center position of the fork tip coincided with the center of the pallet opening, and the pitch angle of the pallet and the tilt angle of the fork coincided. This adjustment was performed by sequentially applying P control using the tilt angle deviation, fork-height deviation, and longitudinal position deviation as inputs.

In the second stage, a straight-line trajectory along the axial direction of the fork was generated from the state shown in Figure 26b, and the fork moved along this trajectory. Specifically, the fork was made to reach out at a constant speed, and after the reach amount reached the limit of the operating range, the vehicle body moved forward at a low speed. During this period, P control was applied to the fork-height deviation to maintain the target trajectory.

In this experiment, considering the size of the pallet and length of the fork (1100 mm × 1100 mm and 1070 mm, respectively), the insertion motion was set to end when the insertion depth reached 900 mm.

### 8.3. Results of Series of Motion Experiments

After calibrating the camera and fork coordinate systems using the improved method described in Section 4 and Section 5, a running test was performed six times. With the fork lifted to 0.85 m, the forklift approached a black pallet placed on a shelf from an initial position and orientation close to X = 3.0 m, Y = −3.0 m, and yaw angle = 110.0°, and inserted the fork. Six running tests were performed, with three successful runs and three failures. The measurement accuracy required for the successful insertion of the forks was analyzed using [12]. The allowable error in the final position and orientation after running in the Y-direction was 50 mm or less, in the yaw orientation was 3° or less, and in the pitch inclination estimation was 1° or less.

#### 8.3.1. First Experiment

**Figure 27:**
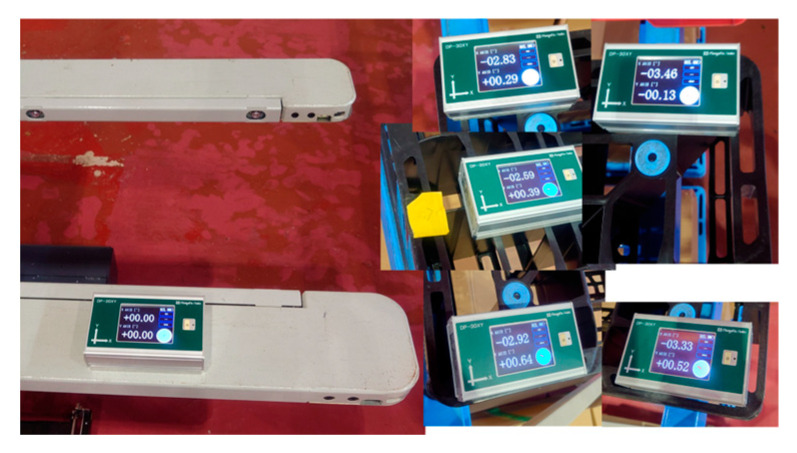
Inclination of pallet measured by inclinometer was approximately −3.026°.

**Figure 28:**
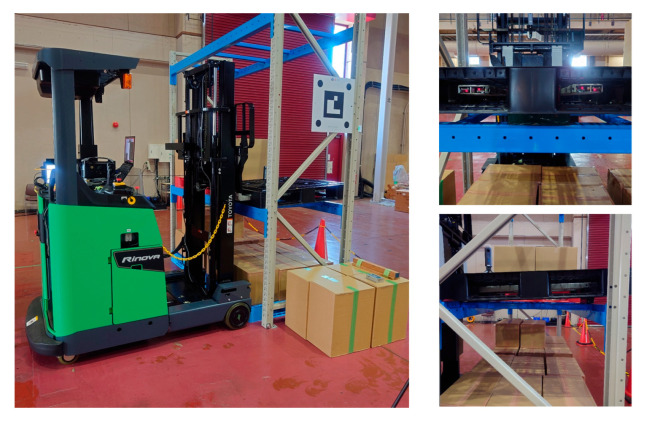
Approach and fork insertion were successful for the first experiment.

**Figure 29:**
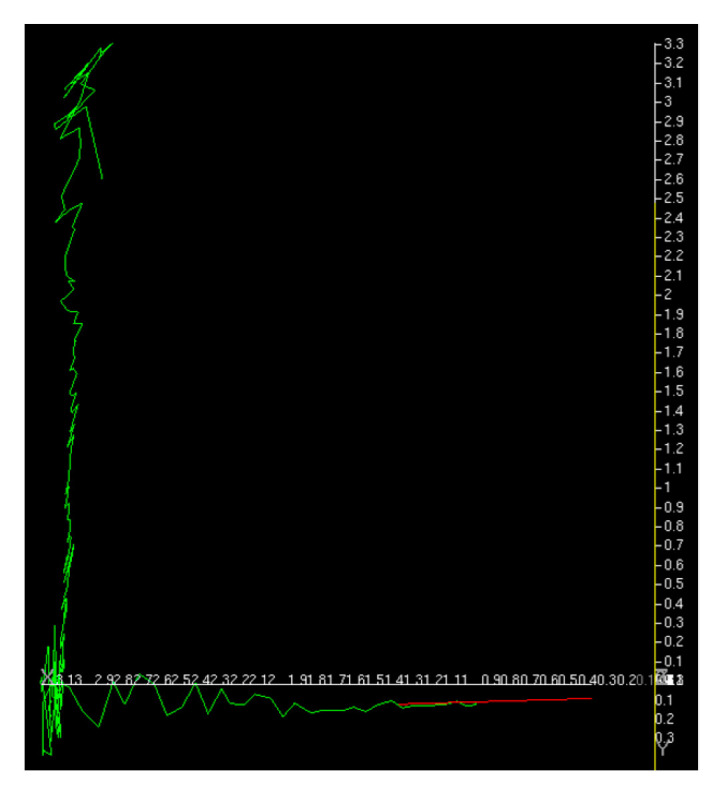
Trajectory of forklift estimated by image measurement was shown in green and the final yaw direction was shown in red for the first experiment.

**Figure 30:**
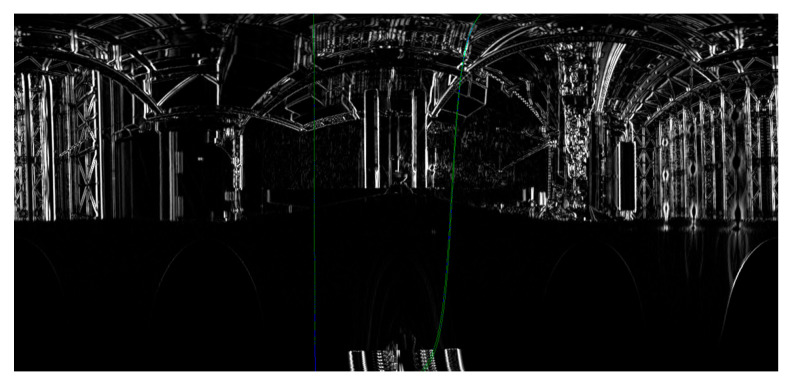
Pitch inclination measurement by detecting straight line (green line) for the first experiment.

The first experiment was conducted without a load by sandwiching square timber behind the pallet. As shown in Figure 27, the inclination of the pallet in the fork coordinate system measured by the inclinometer was approximately −3.026°. The approach, pitch inclination estimation, and fork insertion were successful (Figure 28). The trajectory of the forklift coordinate system estimated by the image measurements is shown in Figure 29. In the pitch inclination measurement, it was judged that there was no load, and the inclination angle was estimated to be −3.3034° from the left and right edges of the upper surface of the pallet (Figure 30). The difference from the measurement by the inclinometer was 0.3° or less.

#### 8.3.2. Second Experiment

**Figure 31:**
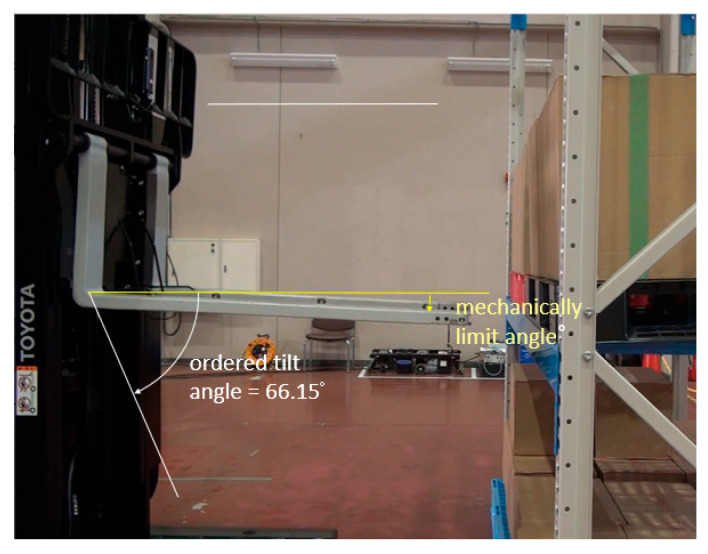
Approach was successful, but fork insertion failed.

**Figure 32:**
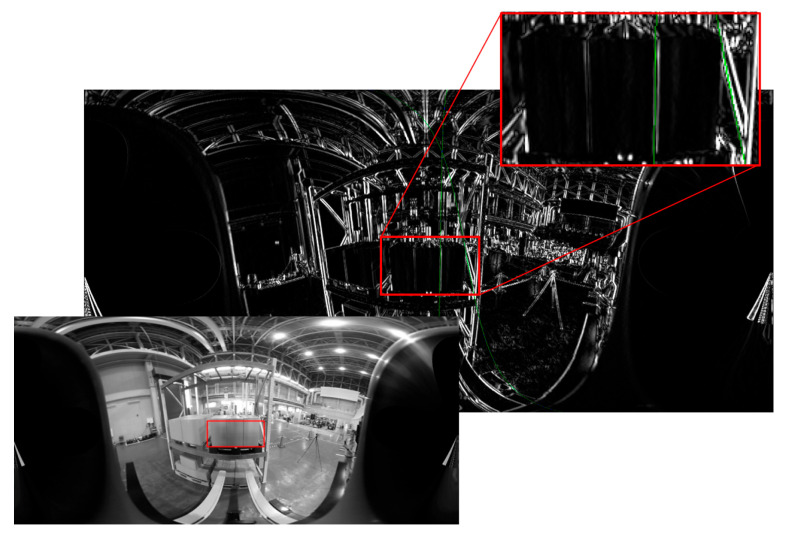
Edge of clamp of shelf was included in detection area shown by red frame.

In the second experiment, cardboard boxes were stacked on a pallet by sandwiching square timber behind the pallet. Although this approach was successful, the fork could not be inserted because the estimated pitch inclination angle was as large as 66.1503, and it exceeded the mechanical limitation of AGF’s fork tilt motion (Figure 31). As shown in Figure 32, because the edge of the shelf clamp was included in the detection area, the edge strength was strong, and the length was sufficient, it was selected as the target of the pitch inclination measurement, which caused large errors.

#### 8.3.3. Third Experiment

**Figure 33:**
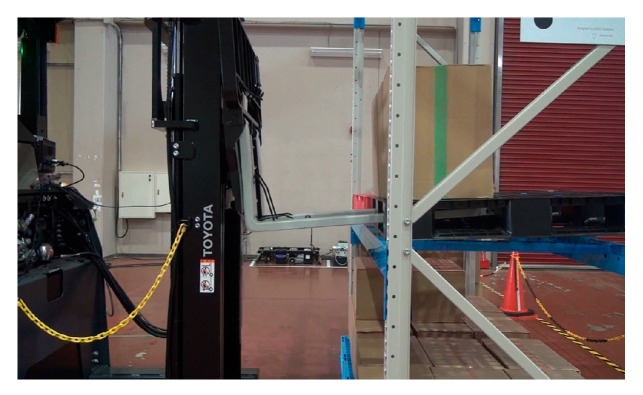
Pallet was pushed when fork was inserted.

**Figure 34:**
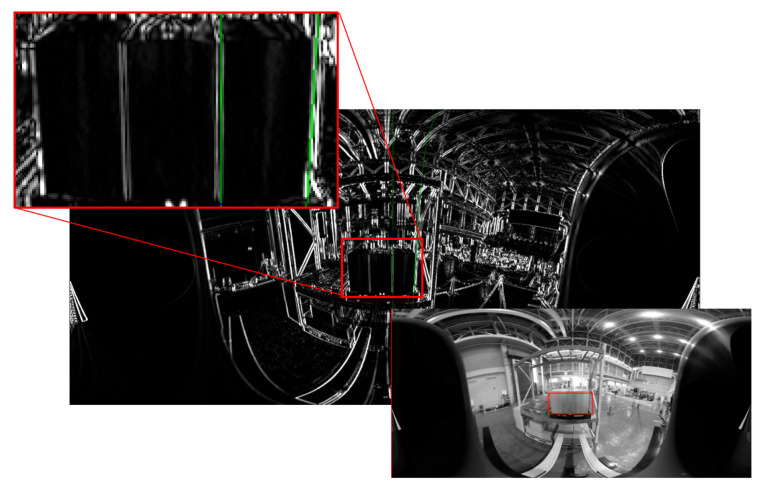
Edge of post on back side of shelf was included in detection area shown by red frame.

The third experiment was conducted with the previous pallet shifted by approximately 5 cm to the left. The approach was successful, but the estimated pitch inclination angle was −6.6935°, and the error was larger than 3°. Therefore, the pallet was pushed when the fork was inserted (Figure 33). The edge of the post on the back side of the shelf was included in the detection area; the edge strength was strong, and the length was sufficient. Therefore, it was selected as the target for the pitch inclination measurement, which caused an error (Figure 34).

#### 8.3.4. Fourth Experiment

**Figure 35:**
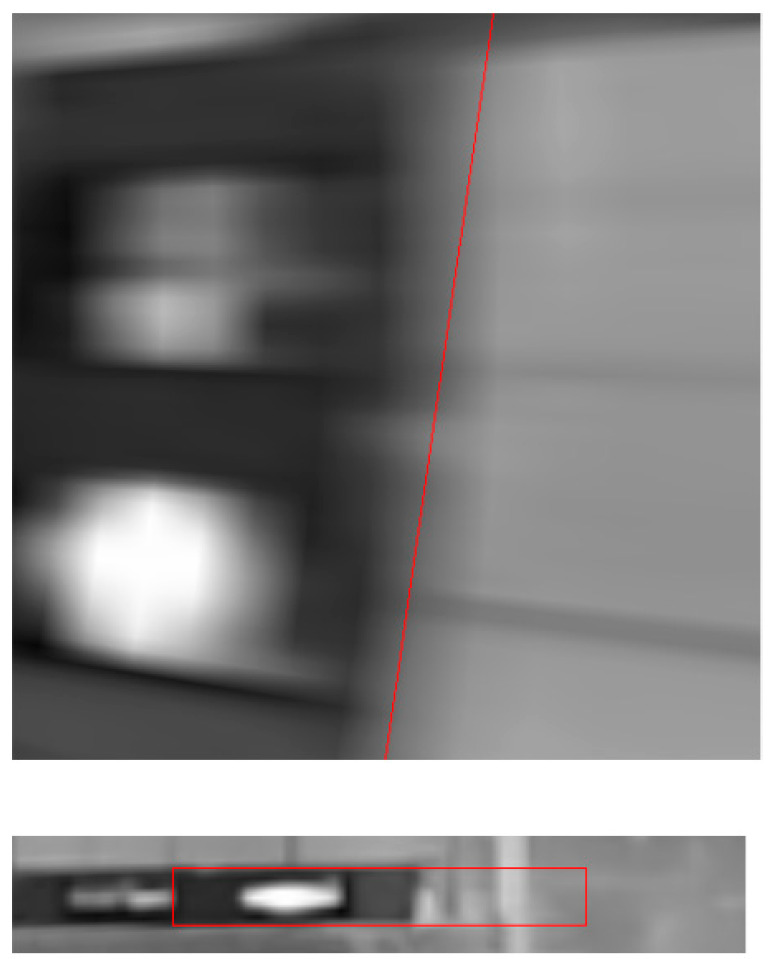
Upper is the horizontal projection plane image and the detected yawline (red line). Lower is the vertical projection plane image with the detected position (red frame) by template matching.

In the fourth experiment, a cardboard box was placed on the pallet such that its width was full, and square timber was sandwiched behind the pallet. At the end of the 90-degree turn at the second waypoint during the approach, template matching in the vertical projection plane image failed, and the position and orientation measurements collapsed and failed (Figure 35). This problem could be solved if the width of the vertical projection plane image for the 90-degree turn at the second waypoint was slightly wider.

#### 8.3.5. Fifth Experiment

**Figure 36:**
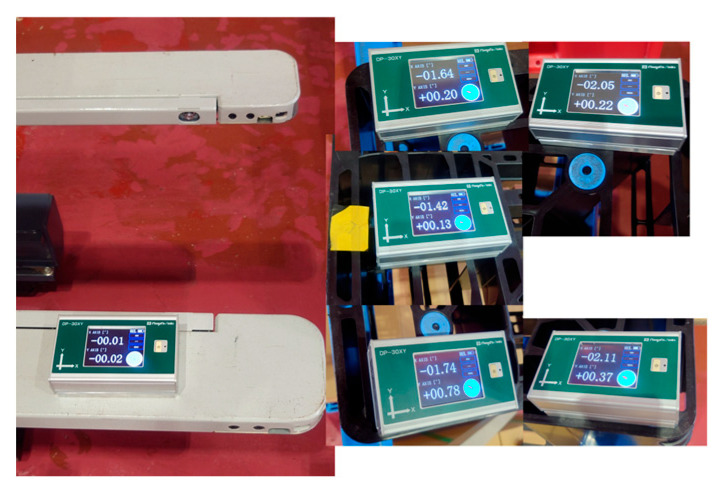
Inclination of pallet is measured by inclinometer.

In the fifth experiment, the control performance of Edge B was adjusted, a piece of paper was inserted behind the pallet, and the experiment was conducted without a load. As shown in Figure 36, the inclination of the pallet in the fork coordinate system was approximately −1.794° as measured by an inclinometer.

**Figure 37:**
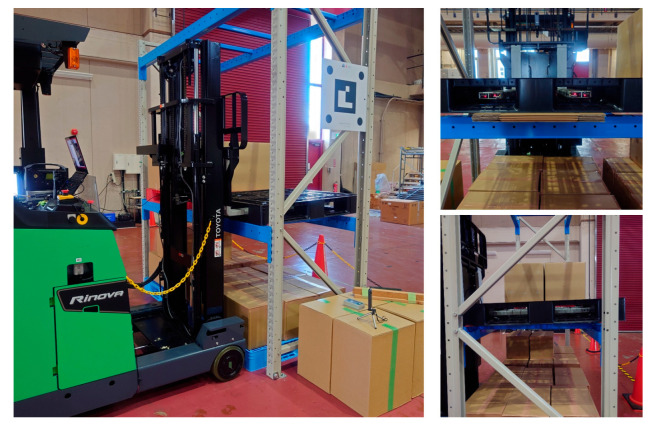
Approach and fork insertion were successful.

**Figure 38:**
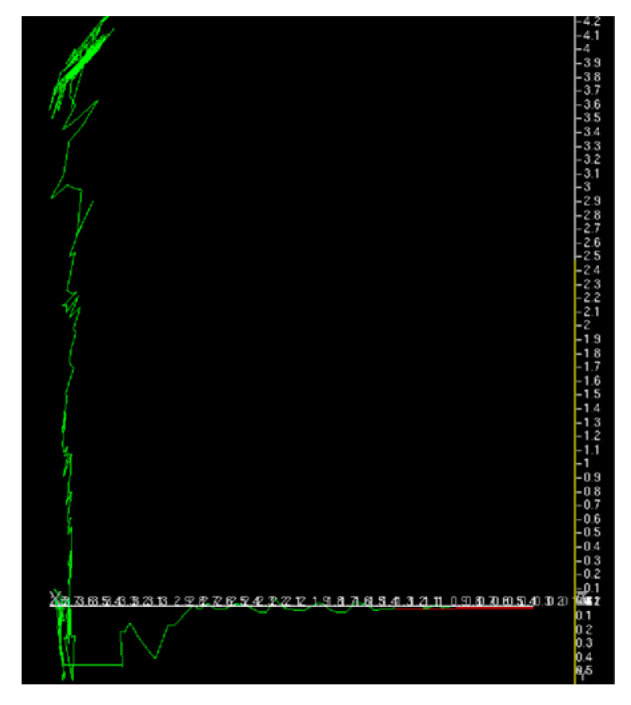
Trajectory of forklift estimated by image measurement was shown in green and the final yaw direction was shown in red for the fifth experiment.

**Figure 39:**
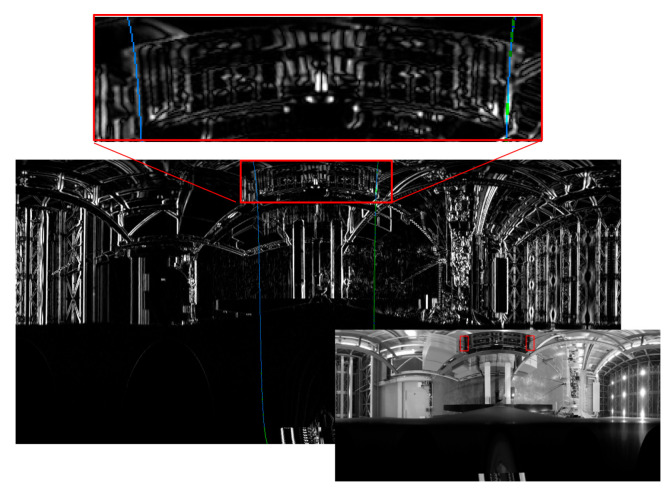
Pitch inclination measurement by detecting straight line (green line) for the fifth experiment.

The approach was successful, the pitch inclination was successfully estimated, and the fork was successfully inserted (Figure 37). As shown in Figure 38, the track of the forklift coordinate system estimated by image measurement improved the follow-up performance to Y = 0 at Edge B. In the pitch inclination measurement, it was judged that there was no load, and the inclination angle was estimated to be −1.65027° from the left and right edges of the pallet’s upper surface (Figure 39). The difference from the measurement by the inclinometer was 0.2° or less.

#### 8.3.6. Sixth Experiment

**Figure 40:**
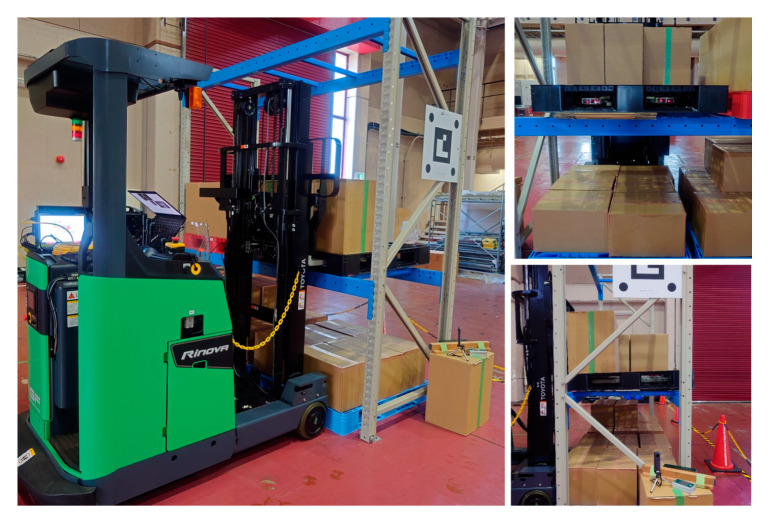
Approach and fork insertion were successful for the sixth experiment.

**Figure 41:**
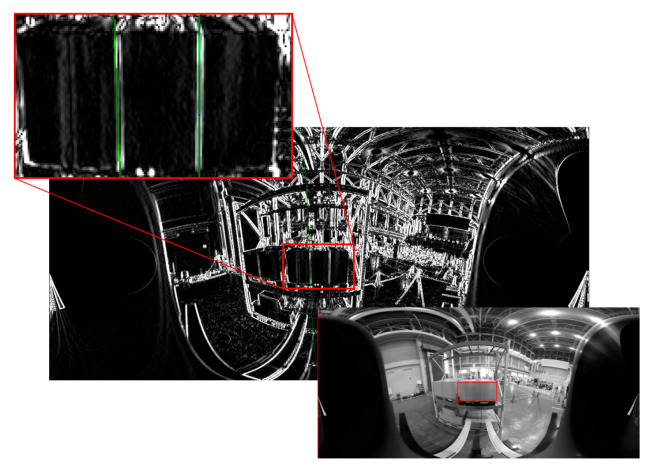
Pitch inclination measurement by detecting straight line (green line) for the sixth experiment.

In the sixth experiment, the paper material was sandwiched behind the pallet, and cardboard boxes were placed such that the width of the pallet was full. The approach, pitch inclination estimation, and fork insertion were successful (Figure 40). In the pitch inclination measurement, it was judged that there was cargo, and the inclination angle was estimated to be −1.18506° from the two edges between the cardboard boxes stacked in three rows (Figure 41). The difference from the measurement by the inclinometer was slightly larger at 0.6° but was sufficiently smaller than the allowable angle for fork insertion.

## 9. Conclusions

By making image processing robust, it was shown that the approach to pallet and fork insertion could be realized in various situations. The six experiments using the actual machine were successful in three experiments with different pitch inclinations, with or without loads. The failure of the pitch inclination measurement resulted from the influence of the edge of the background in the detection area of the load. Although this depends on the situation at the site, failures can be avoided by adjusting the detection area. The failure of the position and orientation measurements during the approach was due to the failure of template matching in the vertical projection plane image while the forklift was turning. This problem can be avoided by adaptively determining the width of the vertical projection plane image from the turning speed of the forklift and its position relative to the pallet. A video of the two successful cases is provided as a Supplement Video S1.

However, when what was originally seen as an edge was no longer perceived as an edge owing to the movement of the viewing position during driving, it was impossible to deal with the situation using image processing alone. Because such situations can occur in the field, it is necessary to continue the operation by utilizing odometry or control commands, even in such cases. It was experimentally verified that this method could be applied when the running surface was uneven. However, there were cases in which the current method assumed that the running surface was flat and could not be applied because of the existence of slopes in the field. In the future, the image-processing algorithm will be improved and implemented so that the application range can be expanded, even when the running surface is inclined, and experimental verification will be conducted.

## Figures and Tables

**Table 1:** Approaches to image measurement methods.

	[Measurement 0]	[Measurement 1-a]	[Measurement 1-b]	[Measurement 2]
[40]	×	◯	×	×
[36]	◯	◯	△	×
[32]	×	◯	△	×
[37]	×	◯	×	×
[34]	×	◯	△	×
[43]	×	◯	×	◯
[39]	×	◯	◯	×
[38]	×	◯	◯	×
[41]	×	◯	◯	×
Presented	◯	◯	◯	◯

**Table 2:** Characteristics of straight lines to be detected from the image.

		Target Image	Possible Appearance Area	Expected Length	Probability of Appearing	Edge Strength
Longitudinal line detection for zenith correction		Panorama image (Z axis center)	No restriction, i.e., the entire image	Not limited: longer is better	Unknown	Random: stronger is better
Longitudinal line detection for calibration		Panorama image (X axis center & Y axis center)	Can be limited	Limitable: For example, 80% of the length of the limited area	100%	They should be strong in the area.
yawline detection		Horizontal projection plane image	Can be limited	Limitable: For example, 80% of the length of the limited area	100%	It should be relatively strong within the area, but the strength will change.
Longitudinal line detection for pitch angle measurement	Unloaded	Panorama image (X axis center)	Can be limited	Limitable: For example, 80% of the length of the limited area	50%	It should be relatively strong in the area, but its strength is unknown.
	Loaded	Panorama image (Z axis center)	Can be limited	Limitable: For example, 80% of the length of the limited area	50%	It should be relatively strong in the area, but its strength is unknown.

**Table 3:** Angle measured by inclinometer (GT) and inclination angle obtained by image measurement (Estimated).

	GT	Before Improvement			After Improvement		
		Esti.	Esti. − GT	(Esti. − Offset) − GT	Esti.	Esti. − GT	(Esti. − Offset) − GT
①	−0.01	0.78	0.79	0.28	0.46	0.48	0.11
②	−2.34	−1.50	0.84	0.33	−1.87	0.47	0.10
③	2.61	3.27	0.65	0.14	3.02	0.41	0.04
④	−3.38	−3.07	0.31	−0.20	−3.12	0.26	−0.11
⑤	−5.91	−5.96	−0.05	−0.56	−5.67	0.24	−0.13
Offset			0.51			0.37	

(unit: degree).

## Data Availability

The original contributions presented in this study are included in the article. Further inquiries can be directed to the corresponding author.

## Supplementary Materials

The following supporting information can be downloaded at: https://www.mdpi.com/article/10.3390/s26010154/s1, Video S1: A video of the two successful cases.

## Appendix A

Summary of robustness and efficacy.

	**Improvement**	**Efficacy**
Measurement 0	Line detection method	Precision up
	Create a panoramic image with a tilted center axis	Precision up
	Use rectangular chess-like panels	Reduces the effects of background and lighting changes
Measurement 1-b	Line detection method	False positive suppression
	Considering edge orientation	False positive suppression
	Compute scale for each input image	Robustness to illumination conditions with viewpoint variations
Measurement 2	Line detection method	False positive suppression
	Compute scale for each input image	Robustness to illumination variation
	Create a panoramic image with a tilted center axis	Precision up
